# Spina Ventosa of the Left Second Metacarpal in an Adult Indian Male With No Pulmonary Involvement: A First-of-Its-Type Case

**DOI:** 10.7759/cureus.58476

**Published:** 2024-04-17

**Authors:** Sankalp Yadav

**Affiliations:** 1 Medicine, Shri Madan Lal Khurana Chest Clinic, New Delhi, IND

**Keywords:** mycobacterium tuberculosis (mtb), cartridge based nucleic acid amplification test (cbnaat), no pulmonary involvement, adult indian male, left second metacarpal, spina ventosa

## Abstract

Tuberculosis inflicting small bones is infrequently reported, even in endemic countries. A case of isolated involvement of the left second metacarpal in an adult Indian male with no pulmonary involvement is rare and has never been documented before in the medical literature. It's a diagnostic challenge due to non-specific clinical features, absence of constitutional signs of tuberculosis, ambiguity on radiograph films at early stages, and often results in delayed diagnosis. Moreover, it's a paucibacillary disease, and hence, diagnosis can be an arduous task. Herein, a case of a 20-year-old Indian male is presented who came with complaints of pain and swelling with a discharging sinus from the dorsum of his left hand. A detailed evaluation with the isolation of *Mycobacterium tuberculosis* on a cartridge-based nucleic acid amplification test helped in the diagnosis and initiation of appropriate antituberculous chemotherapy per his weight.

## Introduction

Tuberculosis, caused by *Mycobacterium tuberculosis*, has long been recognized as a major source of illness and death [1]. It can manifest in either pulmonary or extrapulmonary forms, with the latter accounting for 10-15% of all tuberculosis cases [2]. About 1-5% of all tuberculosis cases and 10-18% of extrapulmonary cases have osteoarticular involvement [3].

Tubercular dactylitis, also known as spina ventosa, refers to tuberculosis affecting the small bones of the hand and foot, such as the metacarpals, metatarsals, and phalanges [4]. This condition shows a preference for the smaller bones of the hand over those of the foot. It is notably less common beyond the age of five, with approximately 85% of cases reported in children younger than six years [5]. Measuring barely one percent of all bone sites, metacarpal tuberculosis is an uncommon manifestation of the disease [6].

A unique case of a 20-year-old Indian male with a profoundly swollen left index finger and purulent discharge is described. Medical treatment was started in accordance with national guidelines after establishing the diagnosis as tuberculosis of the left second metacarpal.

## Case presentation

In the year 2022, a 20-year-old non-diabetic male from a low socioeconomic background came with complaints of painful swelling involving his left index finger for three months. The swelling was small and grew to the size shown below over the last two months. Further, it was associated with a discharging sinus from the base of the left index finger. The discharge was purulent, non-foul-smelling, non-blood-tinged, and yellow in color. He had no history of trauma or any sick contact (including a person with tuberculosis). Also, there was no history of constitutional features of tuberculosis. He was a laborer by occupation with occasional bidi smoking (one in two or three days). He was a slum dweller with no history of imprisonment or stays at refugee camps.

A general examination was suggestive of a young hemodynamically stable man with an ectomorphic build. Additionally, there was no clubbing, icterus, cyanosis, pretibial edema, or pallor. His systemic examination was unremarkable for any disease.

**Figure 1:**
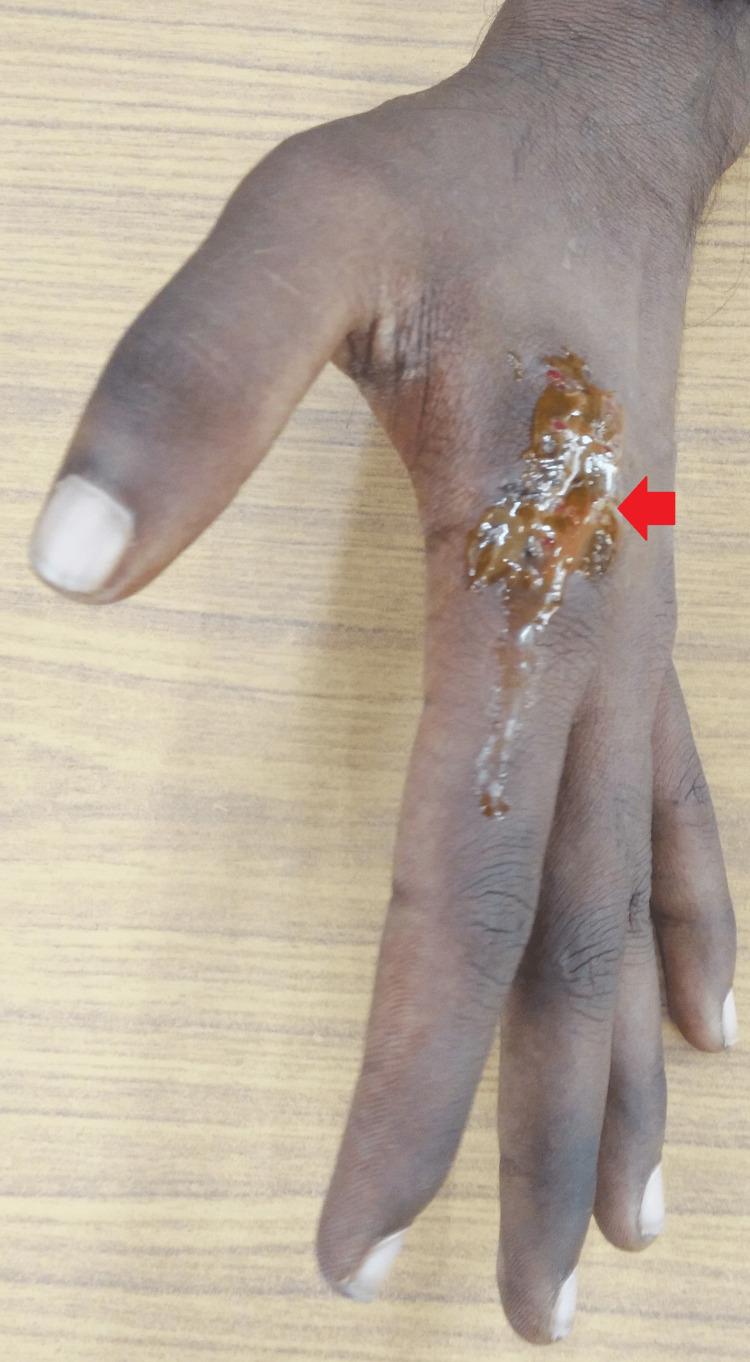
Gross image showing discharging sinus with purulent discharge

A local examination of the left index finger dorsal aspect revealed a swollen base with a discharging sinus about 0.5x0.5 mm near the metacarpophalangeal joint. It was also linked with overlying erythema. The range of motion was restricted at the metacarpophalangeal and proximal interphalangeal joints (Figure 1).

With tuberculous dactylitis, syphilitic dactylitis, and fungal dactylitis as differential diagnoses, a preliminary diagnosis of persistent pyogenic osteomyelitis was made. A diagnostic laboratory workup showed a raised C-reactive protein (9.0 mg/dL), erythrocyte sedimentation rate (64 mm in the first hour), and a strongly positive Mantoux test (18x15 mm induration). The rest of the tests, including HIV, hepatitis panel, rheumatoid factor, induced sputum microscopy, and venereal disease research laboratory tests, were negative. His chest radiograph was not suggestive of any disease.

**Figure 2:**
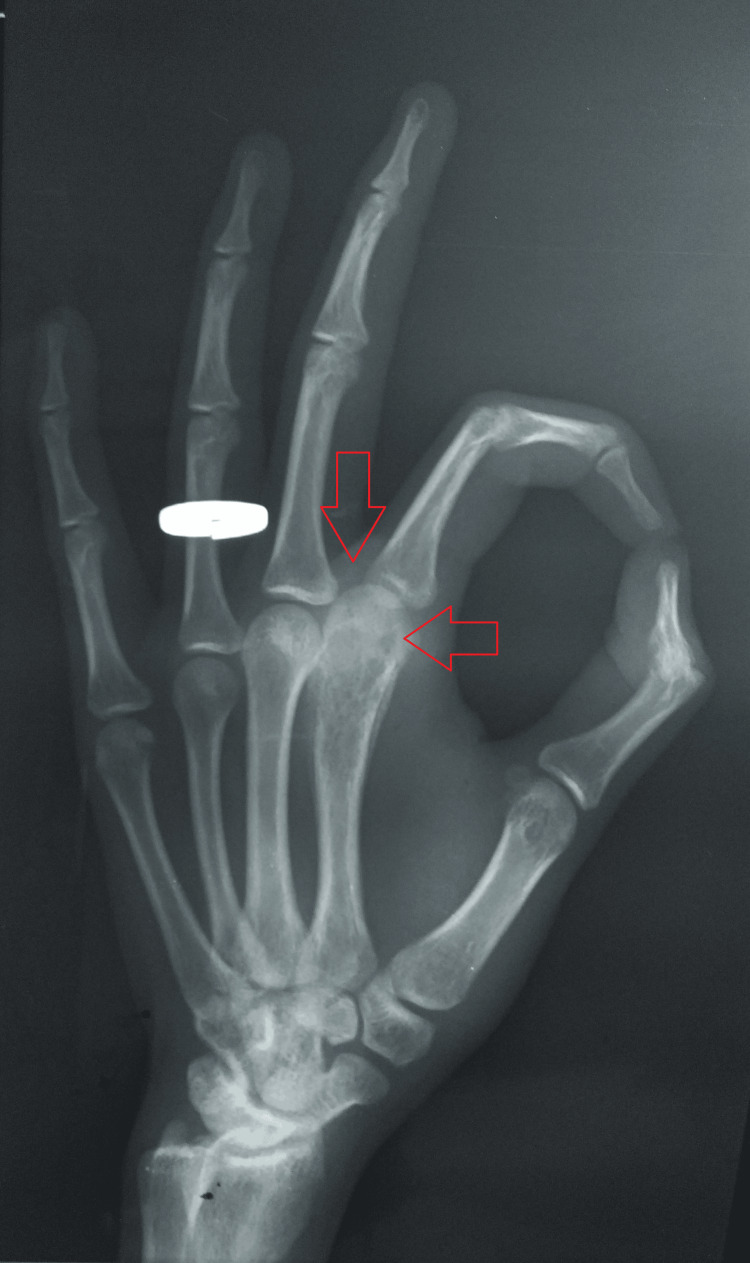
A plain radiograph-oblique view of the hand showing a lytic lesion with slight cortical erosion and minimal periosteal reaction over the head of the second metacarpal of the left index finger

**Figure 3:**
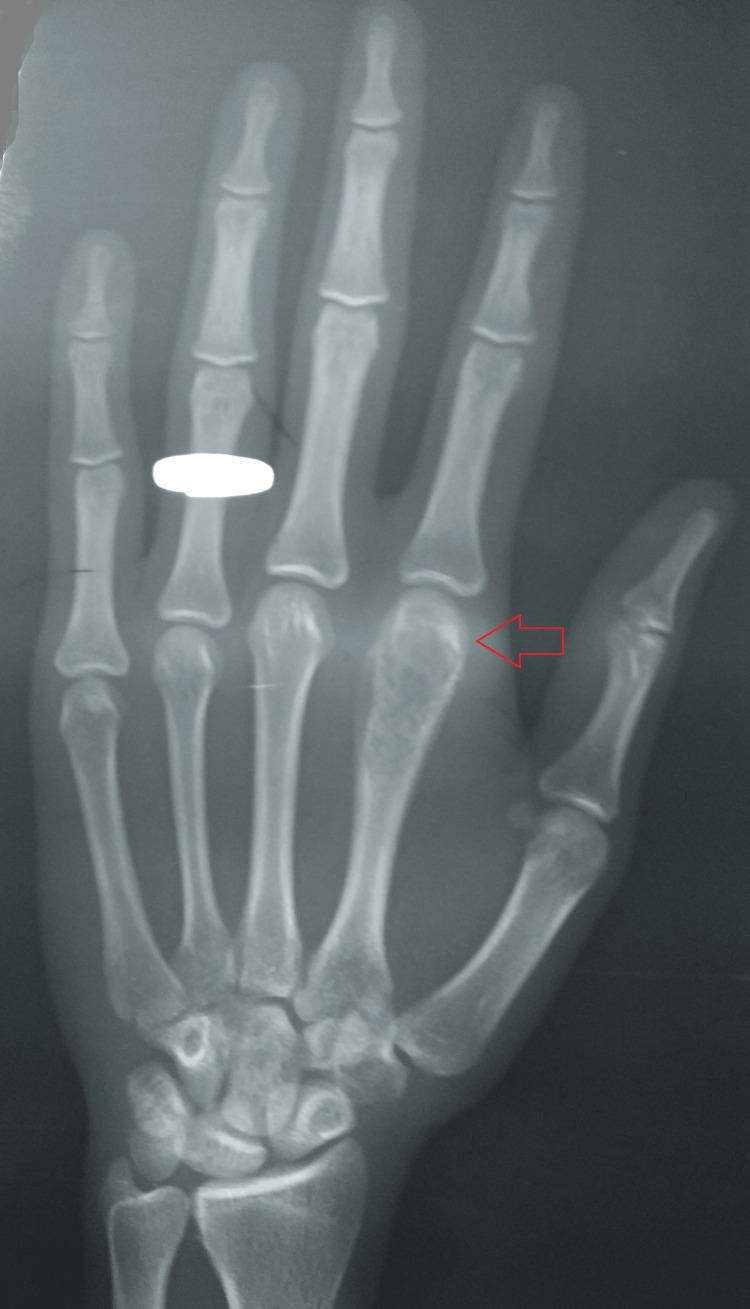
A plain radiograph-AP view of the hand showing a lytic lesion with slight cortical erosion and minimal periosteal reaction over the head of the second metacarpal of the left index finger AP - anteroposterior

A radiograph of the hand was suggestive of a lytic lesion with slight cortical erosion and minimal periosteal reaction over the head of the second metacarpal of the left index finger (Figures 2-3).

After an ultrasound-guided biopsy, granulomatous inflammation involving the dermis in the background of lymphocytes was noticed, along with Langhans giant cells and necrosis. Additionally, samples were sent for gram staining and mycobacterium, fungal, and bacterial culture; nevertheless, the findings were negative. Another sample was sent for examination using cartridge-based nucleic acid amplification. The findings showed that *Mycobacterium tuberculosis *(very low) was present and that it was not resistant to rifampicin.

Eventually, a diagnosis of spina ventosa of the left second metacarpal in an adult male with no pulmonary involvement was made, and antituberculous treatment was started with fixed-dose combinations of isoniazid, rifampicin, ethambutol, and pyrazinamide for two months and then continued with three drugs, i.e., isoniazid, rifampicin, and ethambutol, for the next 10 months.

He was doing fairly well in medical management, with a reduction in swelling, pain, and occasional discharge. There were no major adverse drug reactions. However, after completing two months of antituberculous treatment, he requested a transfer to his village, which was acknowledged. His treatment outcome was mentioned as treatment-complete in the national data portal Nikshay.

## Discussion

Isolated tuberculosis of the metacarpal bones is exceedingly rare, even in areas where the disease is prevalent, and it predominantly affects young individuals (less than six years) and is infrequently reported in adults [7]. Approximately one-third of patients with bone tuberculosis have coexisting active pulmonary disease [6].

Diagnosis of tuberculosis of the metacarpal bones is difficult due to nonspecific appearances on multimodal imaging, overlapping clinical features with other musculoskeletal diseases, the paucibacillary nature of the disease, the absence of constitutional features of tuberculosis in the majority of cases, and a lack of awareness among treating clinicians [5]. Frequently, the initial presentation is soft tissue swelling and periostitis that eventually progresses into expansile bony deterioration and sequestrum development [8].

Pain and swelling have been reported as the most common presenting symptoms [9]. Kotwal et al. reported swelling as the most common clinical feature in a study on 32 patients with hand and wrist tuberculosis [10].

Differential diagnoses include pyogenic osteomyelitis, enchondroma, sickle cell disease, syphilitic involvement, luetic dactylitis, and Brodie's abscess [7,9]. Inflammatory markers and leukocyte counts are often normal, and Mantoux tests are usually positive, as seen in this case; however, a negative reaction does not rule out tuberculosis [9].

Diagnosis is confirmed through histological examination, which reveals caseating giant cell granulomas with epithelioid cells, as seen in the present case [6]. A negative pus culture or failure to detect acid-fast bacilli under the microscope does not exclude tuberculosis [9]. Non-operative or conservative treatment is generally recommended for metacarpal tuberculosis [11]. The majority of authors advocate for a 12-month course of antituberculous chemotherapy, typically involving isoniazid, rifampicin, ethambutol, and pyrazinamide for the first two months, followed by isoniazid, ethambutol, and rifampicin for the remaining 10 months. The same is mentioned in India's national guidelines [11].

To the best of my knowledge and after a thorough medical literature search, this is the first case ever reported of a young adult who presents with isolated second metacarpal tuberculosis without any other findings. Nguyen Ngoc et al. reported the disseminated tuberculous involvement of the second metacarpal in a child; however, their case differs in that he also had pleural effusion and concomitant infection of the metatarsals and phalanges [12]. There was no involvement from any other site in the present case, and this was an adult. Nonetheless, it is important to keep in mind that tuberculosis can manifest in the most peculiar ways and unexpected locations, so clinicians are reminded of the significance of early detection of this uncommon form of an age-old illness. This will help in avoiding any untoward treatment outcomes.

## Conclusions

An exceedingly infrequent case of isolated second metacarpal tuberculosis in an adult male is presented here. The case sheds light on the rare manifestations of a common disease. Even in high-burden settings, such presentations could result in diagnostic delays, ultimately affecting the final results of the management. This case also intends to raise awareness about such presentations among the primary care clinicians where these patients report first. Nevertheless, it was only one case and similar reports should be documented to determine a specific management protocol for such rare clinical entities.
